# Determination of the hyperfine magnetic field in magnetic carbon-based materials: DFT calculations and NMR experiments

**DOI:** 10.1038/srep14761

**Published:** 2015-10-05

**Authors:** Jair C. C. Freitas, Wanderlã L. Scopel, Wendel S. Paz, Leandro V. Bernardes, Francisco E. Cunha-Filho, Carlos Speglich, Fernando M. Araújo-Moreira, Damjan Pelc, Tonči Cvitanić, Miroslav Požek

**Affiliations:** 1 Department of Physics, Federal University of Espírito Santo (UFES), Av. Fernando Ferrari, 514, 29075-910, Vitória, ES, Brazil; 2 Department of Exact Sciences, Federal Fluminense University, 27255-250, Volta Redonda, RJ, Brazil; 3 Department of Physics, Federal University of São Carlos (UFSCar), P.O. Box 676, 13565-905, São Carlos, SP, Brazil; 4 Department of Physics, Faculty of Science, University of Zagreb, Bijenička 32, HR-10000, Zagreb, Croatia

## Abstract

The prospect of carbon-based magnetic materials is of immense fundamental and practical importance, and information on atomic-scale features is required for a better understanding of the mechanisms leading to carbon magnetism. Here we report the first direct detection of the microscopic magnetic field produced at ^13^C nuclei in a ferromagnetic carbon material by zero-field nuclear magnetic resonance (NMR). Electronic structure calculations carried out in nanosized model systems with different classes of structural defects show a similar range of magnetic field values (18–21 T) for all investigated systems, in agreement with the NMR experiments. Our results are strong evidence of the intrinsic nature of defect-induced magnetism in magnetic carbons and establish the magnitude of the hyperfine magnetic field created in the neighbourhood of the defects that lead to magnetic order in these materials.

The occurrence of magnetism in carbon materials has been the subject of many investigations and some controversy along the past two decades, given the enormous interest in the possibility of producing carbon-based magnetic materials free from metallic elements^1^,^2^,^3^. These biocompatible magnetic materials find applications in fields such as drug delivery and magnetic resonance imaging, among others^1^,^4^. Moreover, the design of graphene-based spintronics devices would greatly benefit from a deeper understanding of magnetism and hyperfine interactions in carbon materials^1^,^5^. Recent experimental evidence of magnetic properties (with reports of ferromagnetic order in some cases) of carbon-based materials include irradiated graphite, nanocarbons, fullerenes, oxygen-containing carbons and point defects in graphene^1^,^3^,^4^,^6^,^7^,^8^,^9^,^10^,^11^,^12^,^13^,^14^,^15^. From the theoretical point of view, magnetism in graphene and related materials has been universally associated with the occurrence of defects such as atomic vacancies, chemisorbed species (such as fluorine, hydrogen and oxygen) and edge sites^1^,^3^,^4^,^10^,^12^,^16^,^17^. Similar examples of defect-induced magnetism have also been reported in other materials free from transition metal or rare earth elements, such as organic magnets, oxides, nitrides, silicon carbide and others; in all these cases, a common point is the source of magnetism being related to the spin polarization of *p* orbitals, which is associated with the occurrence of defects of structural or chemical origin^2^,^3^,^18^,^19^,^20^,^21^. In spite of this, there is still some scepticism about the possibility of intrinsic magnetic effects in carbon-based materials, due to the ubiquitously questioned presence of minor amounts of iron or other metallic impurities in experimentally-produced samples that could be the actual source of magnetism^4^,^22^,^23^. However, there are several recent examples of careful analyses that demonstrate in a convincing way the intrinsic nature of magnetism in carbon-derived materials. X-ray magnetic circular dichroism (XMCD) is an element-specific technique that allows the assessment of information about the magnetic moments associated with different elements in the material. XMCD data obtained at the carbon K edge have shown that the magnetization of proton-irradiated samples of carbon films and graphite (as well as for virgin graphite) is indeed associated with the spin polarization of carbon *π* electrons and also with chemisorbed hydrogen^8^,^24^. Similar results were achieved for ion-irradiated SiC crystals, where the ferromagnetic properties of the material were ascribed to electrons in *p* orbitals of atoms in the neighbourhood of atomic vacancies^21^. On the other hand, the use of particle-induced X-ray emission (PIXE) has allowed the determination of elemental contents of common magnetic metals (such as Fe, Ni, Cr, etc.) down to sub-ppm levels in samples of graphite and other carbon materials. With this detailed knowledge about the amounts of impurities, it is now possible to ascertain in many well-documented cases of carbon materials that, even when present, the impurities cannot account alone for the magnetization values and also cannot explain the temperature dependence of the magnetic properties of the analysed materials^3^,^24^,^25^,^26^.

If intrinsic magnetism is indeed a feature of carbon-based materials, then the influence of the local magnetic field on carbon nuclei should be detectable – in the case of systems possessing magnetic order, a strong microscopic field termed the hyperfine field (*B*_*hf*_) at the atomic nuclei is anticipated. Therefore, evidence for *B*_*hf*_ and measurements of its properties are highly desirable for a better understanding of magnetism in carbon-based materials, providing information on the source of magnetism from a local perspective. Besides its importance from a fundamental point of view, the hyperfine interactions are relevant for applications of graphene and related materials in spintronics and quantum information processing^27^,^28^,^29^,^30^, leading to numerous theoretical calculations^27^,^28^,^31^ and experimental investigations involving the use of different techniques – electron spin resonance (ESR)^11^, muon spin rotation (*μ*SR)^32^ and perturbed angular distribution (PAD)^33^. In none of these reports, however, any clue about the *B*_*hf*_ value in a truly ferromagnetic carbon material was ever reported. In this work we present a direct measurement of the local magnetic field using ^13^C nuclear magnetic resonance (NMR), corroborating the intrinsic nature of carbon magnetism and comparing the results to DFT calculations, confirming that the magnetism originates from defects in the structure, and not from ferromagnetic impurities.

## Results

### Experimental approach

As a sensitive probe of local magnetic fields, nuclear magnetic resonance is ideally suited for gaining information on the hyperfine field in magnetic carbon-based materials. In NMR experiments, nuclear spin transitions between energy levels are excited and detected, with the transition frequency being characteristic of the type of nucleus and dependent on the local magnetic field according to:


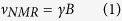


where *γ* is referred to as the gyromagnetic ratio of the studied nucleus (in our case ^13^C) and *B* is the *net* magnetic field at the nucleus site. Local, internal magnetic fields thus contribute to the transition frequency, but in the majority of cases these fields are tiny, so that an additional large external field is necessary to perform the experiment. The net field is then 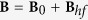, with **B**_0_ the external field and **B**_*hf*_ the local hyperfine field. However, in a magnetically ordered material the local field itself is large enough to enable the experiment to be performed without external field. This is referred to as zero-field NMR, and is a well-known effect in ferromagnetic materials such as iron, nickel and cobalt^34^,^35^,^36^. In the case of zero-field NMR the net field is simply the hyperfine field **B**_*hf*_, and the NMR frequency is proportional to it. Thus a detection of a zero-field ^13^C NMR signal provides direct evidence of intrinsic carbon magnetism and presents a straightforward method of measuring the hyperfine field.

Another important feature of NMR in magnetic materials is a strong signal enhancement due to the coupling of nuclear and (ferromagnetically ordered) electronic spins. This ferromagnetic enhancement is largest in magnetic domain walls^37^, meaning that in zero-field NMR experiments one usually observes the signals from nuclei in the walls. The enhancement can be measured easily if the pulsed NMR method is employed for detecting the zero-field NMR signal. In pulsed NMR, short high power radio-frequency pulses are used to rotate the nuclear magnetization, and the subsequent magnetization precession is detected. In conventional single-pulse NMR experiments performed in non-magnetic materials, the signal amplitude is


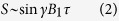


where *B*_1_ is the amplitude of the radio-frequency field, and *τ* the duration of the pulse. For more complex pulse sequences the equivalent expressions become more involved, but the dependence on *γB*_1_*τ* remains qualitatively similar. However, ferromagnetic enhancement increases the field seen by the nuclei in a single domain wall by a factor *η*: *B*_1_ → *ηB*_1_, implying that a measurement of the amplitude in dependence on *B*_1_ can yield the value of *η* and provide insight into domain wall physics. Usually simple relations similar to equation (2) are not obeyed in ferromagnetic powders because of the random orientations of domain walls; more complicated dependences of the signal on *B*_1_ are then observed.

The material chosen for this investigation was ferromagnetic graphite, produced by controlled oxidation of high-purity graphite (details about sample preparation are given in Methods and Supplemental Material). As previously described^13^,^14^,^38^, this material can be produced in bulk quantities (~50 mg) and presents ferromagnetic order at room temperature and below. The maximum amounts of metallic impurities detected in the samples are well below the limits required to account for its overall magnetization, pointing to a genuinely carbon-originated magnetism^14^. (See also Supplemental material.) The magnetic properties of the material are related to the defects introduced in the graphite lattice by the oxygen attack, as suggested by recent theoretical calculations performed in graphite nanoribbons with edges partially passivated by oxygen atoms^12^. The sample selected for the NMR experiments showed a well-defined hysteresis loop in a magnetization versus applied magnetic field measurement conducted at low temperature (1.8 K), with coercive field of ca. 500 Oe, clearly indicating its ferromagnetic character (Supplemental Figure S1).

### Electronic structure calculations

**Figure 1:**
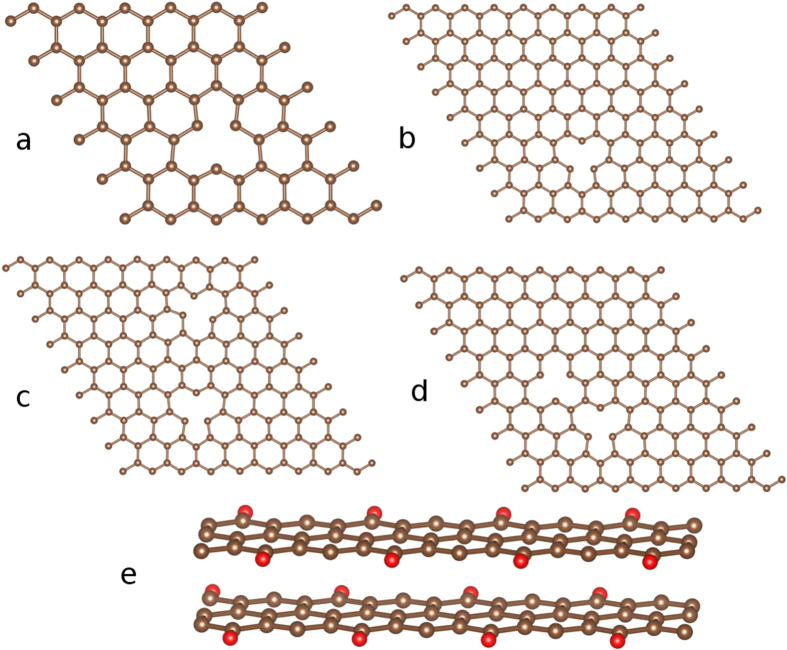
Relaxed structures of the systems used in the DFT calculations: (**a**) Graphene sheet with one single vacancy: supercell with 71 carbon atoms and 1 atomic vacancy. (**b**) Graphene sheet with one single vacancy: Supercell with 161 carbon atoms and 1 atomic vacancy. (**c**) Graphene sheet with two single vacancies: Supercell with 160 carbon atoms and 2 atomic vacancies ca. 11 Å apart. (**d**) Graphene sheet with two single vacancies: Supercell with 160 carbon atoms and 2 atomic vacancies ca. 4.5 Å apart. (**e**) Graphite nanoribbon: Supercell with 48 carbon atoms (indicated as brown spheres) in each layer, with 8 oxygen atoms (indicated as red spheres) at the zigzag edges.

**Figure 2:**
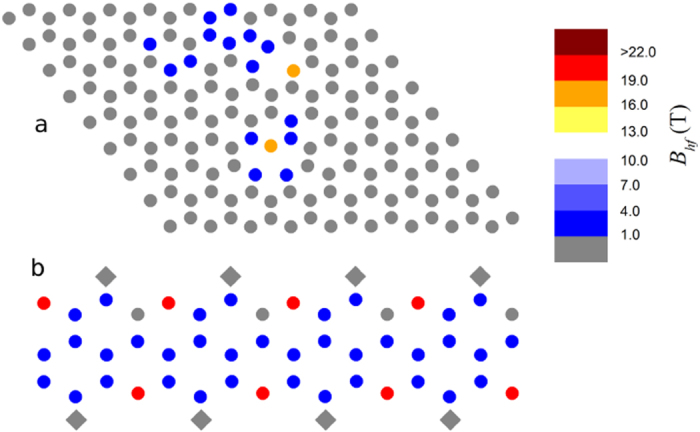
Colormaps showing the distribution of calculated *B*_*hf*_ values. (a) for system C (graphene sheet with two atomic vacancies ca. 11 Å), and (b) along a single graphene layer in system E (graphite nanoribbon). The circles represent carbon atoms and the diamonds in (**b**) represent oxygen atoms.

Without any *a priori* information about the hyperfine field, it would be difficult to find the zero-field nuclear resonance signal. Thus a series of first-principles calculations based on the density functional theory (DFT) was carried out to guide the NMR experiment. Calculations were performed in nanosized model systems built to somewhat reproduce the local features of the structure of ferromagnetic graphite (Fig. 1) (details of the DFT calculations are given in the Methods section). The model systems included graphene sheets with isolated or multiple single atomic vacancies, as well as graphite nanoribbons with oxygen atoms adsorbed at the zigzag edge sites. These systems are known from previous reports^4^,^9^,^10^,^11^,^12^,^16^,^17^,^39^ to give rise to magnetic moments localized at carbon atoms, with indications of a ferromagnetic ground state in some cases^11^,^12^,^39^, and thus they were considered good candidates for the initial calculations of *B*_*hf*_ at ^13^C nuclei. A summary of the results of the DFT calculations is presented in Table 1. The first noticeable aspect of these results is that all *B*_*hf*_ values fall into the same range, ~18–21 T, despite the different types of defects giving rise to magnetism in vacancy-containing graphene sheets and in oxygen-containing graphite nanoribbons. This is an indication that the *B*_*hf*_ values here reported are indeed characteristic of carbon sites with localized magnetic moments in carbon-based systems. As it should be expected, the largest *B*_*hf*_ values in each system were found at the sites also presenting the highest net spin densities and, thus, the largest atomic magnetic moments, as illustrated in Fig. 2 for some of the studied systems.

There are some variations in the average *B*_*hf*_ values as well as in the total magnetic moments of each system due to the possible interactions between neighbour atomic magnetic moments. As an example, the comparison between systems containing a single atomic vacancy (systems A and B, as described in Table 1 and with supercells exhibited in Fig. 1) shows a slightly reduced *B*_*hf*_ value for the larger supercell, corresponding to the larger separation between each defect and its image produced by the use of periodic boundary conditions in the calculations. Similarly, in the case of two interacting single vacancies (systems C and D), the *B*_*hf*_ value and the total magnetic moment were found to increase with the reduction in the separation between the two ferromagnetically coupled vacancies. The largest total magnetic moments and *B*_*hf*_ values among the studied systems were found for the graphite nanoribbon, where the magnetic response is the result of the ferromagnetic coupling between the magnetic moments associated with dangling bonds at edge sites^12^.

It is important to stress here that none of these models is supposed to describe in detail the structural features of bulk ferromagnetic graphite. It is clear that the details about the structure and the mechanism leading to the emergence of the magnetic properties in this material remain to be elucidated, in spite of previous insights about the role played by oxygen-induced defects^13^,^14^. The use of the graphite nanoribbon with oxygen atoms at the edges in the DFT calculations is simply one of many possibilities to model an oxygen-containing graphitic system having structural defects and presenting a ferromagnetic ground state, as previously shown by theoretical calculations^12^. However, the results of the DFT calculations show that irrespective of the specific structural defect present in each one of the modeled systems, the local magnetic moment and the hyperfine magnetic field remain nearly constant. Thus, the present calculations are thought to represent well the local neighbourhood of structural defects in ferromagnetic carbon materials, at least from the point of view of the hyperfine magnetic coupling.

### NMR measurements and comparison to calculations

Guided by DFT calculations, zero-field NMR experiments were carried out in a frequency range encompassing the resonance frequencies corresponding to the predicted *B*_*hf*_ values.

**Figure 3:**
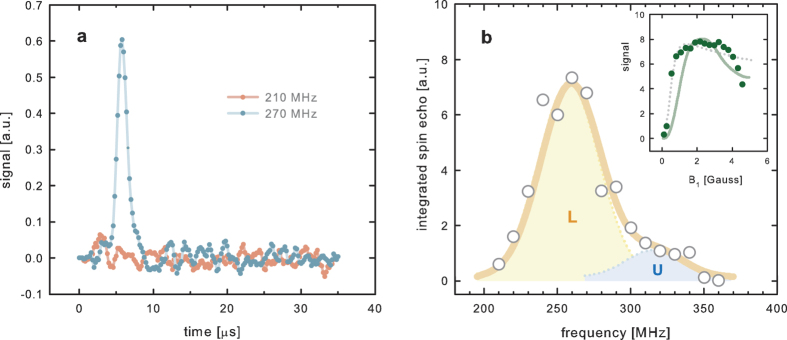
Zero-field ^13^C NMR in ferromagnetic carbon. (**a**) Raw time-domain zero-field NMR signal due to ^13^C nuclei in ferromagnetic graphite at two frequencies (red circles 210 MHz, blue circles 270 MHz). The spin echo is clearly visible at 270 MHz, whereas the ‘blank’ measurement at 210 MHz demonstrates the absence of spurious baseline signals. (**b**) ^13^C zero-field NMR spectrum, obtained by integrating spin echoes at different frequencies, at 1.5 K. Two distinct NMR lines are resolved, the faint upper (U) and much stronger lower (L) line (solid line is a double Gaussian fit). Inset shows the dependence of the L line signal intensity on the excitation pulse field *B*_1_, with strong ferromagnetic enhancement effects visible. Dotted line is the drumhead model of domain wall dynamics, and solid line is an anisotropic model (see text and Supplementary material).

Taking the predicted hyperfine field from DFT data (Table 1), using the proportionality between nuclear magnetic resonance frequency and hyperfine field, *ν*_zfNMR_ = *γB*_hf_, and the well-known magnetogyric ratio of ^13^C (*γ* = 10.569 MHz/T)^40^, the resonance frequencies were estimated to be in the range 200–230 MHz. A significant zero-field NMR signal was indeed found at frequencies just above this range at 1.5 K by sweeping the frequency. We employed the standard two-pulse NMR technique known as spin echoes^41^, where specific radio-frequency pulses are used to refocus the nuclear magnetization at a moment in time (Fig. 3a). The spin echo signal is unique of magnetic resonance, eliminating possible artefacts (as clearly demonstrated with the ‘blank’ measurement at 210 MHz). The maximum signal was detected at ca. 260 MHz, as shown in the NMR spectrum exhibited in Fig. 3b. The *B*_*hf*_ value corresponding to this peak is around 24 T. The deviation from the calculated values given in Table 1 is not surprising, considering the complexity of the real material (from the chemical, structural and magnetic point of view) in comparison to the idealized model systems used in the DFT calculations. The sizeable width of the zero-field NMR line indicates that a hyperfine field distribution exists within the material, which is also qualitatively in agreement with calculations.

The zero-field NMR spectrum shown in Fig. 3b was fitted with two Gaussian lines, named lower (L) and upper (U) lines, with different intensities (L being thus the dominant contribution in zero-field NMR spectra). The two lines may originate from sites with different local fields – corresponding to different types of defects – or from nuclei in different domain wall environments. Strong ferromagnetic enhancement effects are visible in the dependence of the L peak intensity on RF pulse amplitude *B*_1_ (Fig. 3b, inset) – a rough comparison to equation (2) yields an enhancement factor of *η* ~ 1000, somewhat smaller than the values in well-known ferromagnetic materials such as iron and nickel (where *η* ~ 10^4^ in domain walls). This is additional evidence that the observed spectrum is indeed associated with the ferromagnetic-enhanced response of ^13^C nuclei in ferromagnetic graphite. However, the precise shape of the *B*_1_ dependence is rather unusual and cannot be exactly reproduced within commonly accepted models of ferromagnetic NMR enhancement. The two lines in the inset of Fig. 3b are the ‘drumhead’ model of NMR enhancement in domain walls (modeling the domain wall motion in the applied RF field as vibrating drumheads^42^ – dotted line) and a model with strong domain wall pinning anisotropy (due to the graphene-like layers in the graphite structure – full line). Domain wall dynamics in ferromagnetic graphite is thus interesting in itself, warranting further investigation (for more details on the enhancement models see Supplementary Information).

**Figure 4:**
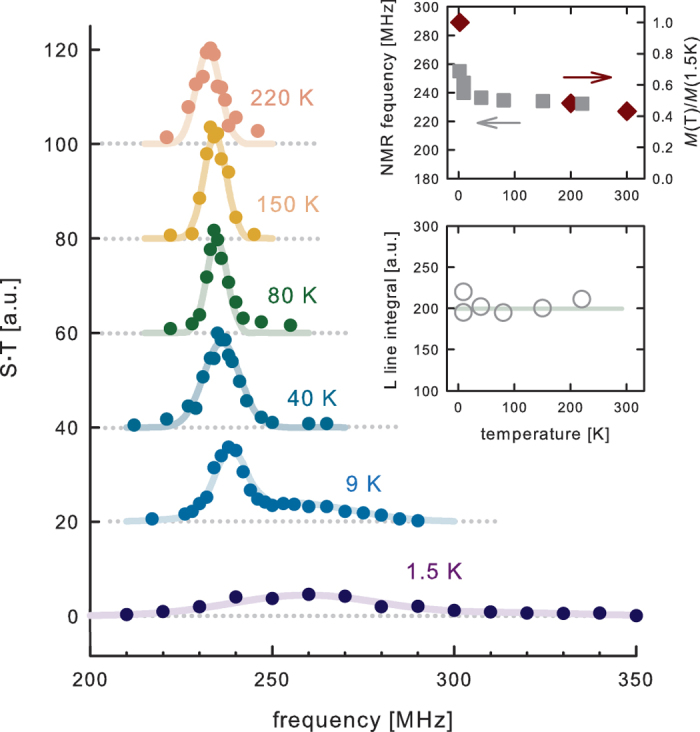
Temperature dependence of the zero-field NMR spectrum of ferromagnetic graphite. Signal intensity is multiplied by temperature to compensate for the usual Boltzmann factor. The broadened low-temperature line morphs into a sharper signal, which remains approximately the same up to 220 K. No signals were detected at 300 K at frequencies from ~180 to ~300 MHz. The spectra are direct evidence of the connection between zero-field NMR and the high-temperature magnetism previously observed in ferromagnetic graphite. Upper inset: comparison of the L line NMR frequency and saturation magnetization normalized to the low-temperature value (from ref. 13). The high-temperature behaviour is the same, but the magnetization at low temperatures grows at a larger rate as compared to the increase in local field seen in NMR. The probable reason is the existence of additional contributions from different magnetic sites (as evidenced by the appearance of the upper NMR line). Lower inset: integrals of the L lines from the main figure, in dependence on temperature. Its constancy implies that the low-temperature shifted and broadened line originates from the same nuclei as the high-temperature signal.

A zero-field carbon NMR signal was also detected at temperatures significantly higher than 1.5 K (Fig. 4), establishing a correspondence with the high-temperature ferromagnetism observed previously in ferromagnetic graphite^13^,^14^. Interestingly, the L line narrows and shifts to lower frequency above ~10 K. This is indication of glassy magnetic dynamics at low temperatures; such effects are not uncommon in ferromagnetic NMR^43^,^44^,^45^ and ought to be expected in a strongly disordered material (from the magnetic viewpoint) like ferromagnetic graphite. The high-frequency U line was only observed at the lowest temperatures, most probably due to fast spin-spin relaxation, making the signal undetectable at higher temperatures. In zero-field NMR, the hyperfine magnetic field (and thus the NMR frequency) is expected to change proportionally to the saturation magnetization as a function of the temperature. Previous reports on the magnetic behavior of ferromagnetic graphite show that the magnetization does not decrease significantly from 2 K up to 300 K, and magnetism is still present at 350 K^13^,^14^. The same general trend was indeed observed for the zero-field NMR frequency of the L line up to 220 K (Fig. 4, upper inset), with a noticeable agreement especially at high temperatures. This is also in line with the constancy of L line intensity (Fig. 4, lower inset). Thus, it is clear from these data that the NMR signal and the magnetic response are likely to be produced by the same source (i.e., structural defects). The failure to detect the U line at high temperatures makes difficult a more detailed direct comparison of microscopic NMR data and macroscopic sample magnetization. Namely, the saturation magnetic moment of the sample is proportional to the local magnetization (and thus to the local field) multiplied by the volume fraction of magnetized material. The NMR signal intensity is a measure of the volume fraction, and thus the unknown U line contribution renders a quantitative comparison impossible. The extra magnetization at low temperatures is then plausibly related to the U line signal. An NMR signal above 220 K was probably not observed due to spin-spin and spin-lattice relaxation effects, since fluctuations increase as the Curie temperature is approached.

## Discussion

The detection of a zero-field NMR signal is unambiguous evidence of the intrinsic nature of carbon magnetism, since the local field at the carbon sites would on average be very small if the magnetism was concentrated in microscopic ferromagnetic impurities. The possibility that the observed signal is not from ^13^C, but ^57^Fe in iron impurities, can be safely discarded: due to the small magnetogyric ratio of ^57^Fe, the local field at the Fe sites would amount to an unrealistic value of ~200 T. The concentration of other possible magnetic impurities in the samples is vanishingly small, but even if there are some minor impurities, they cannot be the cause of the detected NMR signal. For example, the ^57^Fe NMR signal in metallic iron would appear at 45 MHz, in iron oxides at 60–80 MHz and in iron carbides at 30 MHz. The observed NMR signal is also inconsistent with known hyperfine magnetic fields of nickel, cobalt and many alloys^37^,^41^,^42^. Also, transferred fields can be eliminated: a simple calculation of the transferred field at ^13^C nuclei in ferromagnetic cementite (Fe_3_C) yields values at least an order of magnitude smaller than the *B*_*hf*_ detected in the NMR experiments.

Our results also strongly suggest that an all-important role is played by defects in the structure of ferromagnetic graphite, corroborating previous findings on similar materials^3^,^24^,^25^. Most importantly, these defects cause the magnetism on a microscopic level, as evidenced by the good agreement between calculated and measured local magnetic fields. But the strongly disordered structure also gives rise to unconventional domain wall dynamics – as observed in the dependence of signal intensity on RF field amplitude in the NMR experiments – and a broadening of the NMR line at low temperatures. An NMR signal was observed up to 220 K, establishing a correspondence between the zero-field NMR discussed here and the previously detected high-temperature magnetism in carbons.

It is worth noting that the features of the NMR spectrum shown in Fig. 3 were well reproduced in an independent experiment performed with a second ferromagnetic graphite sample from another batch (see Supplemental Material), confirming the robustness of the method of sample preparation and the intrinsic character of the zero-field NMR signal.

To conclude, ^13^C zero-field NMR experiments allowed us to directly evaluate the local hyperfine magnetic field in a ferromagnetic carbon-based material for the first time, corroborating the intrinsic nature of the magnetism. A comparison of the experimental hyperfine fields to DFT calculations shows reasonable agreement, supporting the view that magnetism originates from various defects in the material structure. Thus our results significantly contribute to the understanding of defect-induced magnetic order in carbon materials and open the possibility of more in-depth studies of carbon-based magnetism.

## Methods

### Samples

The samples of powder ferromagnetic graphite were obtained by a chemical route consisting of a controlled etching on the synthetic graphite structure by a vapour phase *redox* reaction in closed argon atmosphere (Ar, 1 atm.) with copper oxide (CuO)^13^,^14^,^38^. For details on the synthesis see Supplementary Material. The chemical, structural and magnetic properties of all analysed samples of ferromagnetic graphite were verified by different techniques^13^,^14^, including atomic absorption spectroscopy (AAS), X-ray fluorescence analysis, energy dispersive spectroscopy, X-ray diffraction, scanning electron microscopy, atomic and magnetic force microscopy, and SQUID magnetization measurements as a function of absolute temperature and applied magnetic field. The average metal concentrations determined by AAS for samples prepared using the same experimental method in different batches are (in weight ratios): Fe – 64 ppm; Co – 0.2 ppm; Ni – 1.2 ppm; Cu – 3.5 ppm; Zn – 1.0 ppm. As mentioned above, these values are too small to account for the sample saturation magnetization observed at low temperature. For example, in the most unfavorable situation (from the point of view of impurity-originated magnetism), all Fe content would be associated with ferromagnetic metallic Fe aggregates or Fe_3_O_4_ particles (which is quite unlikely). Even in this case, the Fe content in this sample would lead to a saturation magnetization at low temperature around 0.01 or 0.006 emu/g, respectively. These values are orders of magnitude below the observed saturation magnetization (Supplemental Figure S1). It is thus clear that the magnetic properties of ferromagnetic graphite are not due to impurities, similarly to what has been reported for other types of magnetic carbons^3^,^24^,^25^.

### DFT calculations

The five systems shown in Fig. 1 were investigated using density functional theory (DFT). In the case of system E, the 3-dimensional structure of the graphite nanoribbon was generated by using periodic boundary conditions along the direction perpendicular to the layers. A full structural optimization of all studied systems was performed. For the exchange correlation potential, the generalized-gradient approximation (GGA) in the Perdew-Burke-Ernzerhof (PBE) scheme^46^ was used, as implemented in the VASP code^47^,^48^. The lattice parameter for graphene obtained from structural optimization was 2.46 Å. All calculations were carried out with spin-polarization and with a plane-wave-cutoff energy of 400 eV; the Brillouin zone integration was performed using the Monkhorst-Pack scheme with a 5 × 5 × 1 k-point mesh^49^. The lattice parameter was kept fixed at the calculated value, whereas the atoms were allowed to relax until the atomic forces were smaller than 0.025 eV/Å. From the relaxed atomic structure of the studied systems, further DFT calculations were conducted to determine the hyperfine magnetic fields, using the all-electron full-potential linearized augmented plane-wave plus local-orbital method as implemented in the WIEN2k package^50^. The muffin-tin radii for carbon and oxygen atoms were selected as RMT = 1.25. The cutoff wavevector RMTKMAX was taken to be 7.0. Self-consistency of the calculations was achieved with energy and charge convergence criteria set to 0.01 *m*Ry and 0.0001 *e*, respectively.

### NMR experiments

NMR experiments were performed using a Tecmag Apollo pulsed NMR spectrometer and an Oxford Instruments high-homogeneity variable field superconducting magnet with a helium variable temperature insert. NMR spectra were acquired with a conventional Hahn echo pulse sequence^41^, *π*/2-*τ*-*π*-echo with a constant echo delay of either 13 *μ*s or 5 *μ*s (for measurements above 1.5 K). The length of the ‘optimal’ *π*/2 pulse was found to be ambiguous due to RF enhancement effects, so a fixed pulse length of 0.5 *μ*s was chosen for all experiments. The sample and the NMR coil were immersed in a liquid-gas helium cryostat at atmospheric pressure.

## Additional Information

**How to cite this article**: Freitas, J. C. C. *et al.* Determination of the hyperfine magnetic field in magnetic carbon-based materials: DFT calculations and NMR experiments. *Sci. Rep.* **5**, 14761; doi: 10.1038/srep14761 (2015).

## Supplementary Material

Supplementary Information

## Figures and Tables

**Table 1:** Calculated hyperfine magnetic field (*B*_*hf*_) in ferromagnetic carbon-based systems.

**System**	***B*_*hf*_(T)**
Graphene sheet with one single vacancy (System A)	19.4
Graphene sheet with one single vacancy (System B)	18.8
Graphene sheet with two single vacancies (System C)	18.3
Graphene sheet with two single vacancies (System D)	20.2
Graphite nanoribbon (System E)	20.8

The reported values correspond to the average taken over the sites where the largest magnetic moments were located in each system.
