# Supplementary material for: Determination of the hyperfine magnetic field in magnetic carbon-based materials: DFT calculations and NMR experiments
Source: Sci Rep. 2015 Oct 5;5:14761. doi: 10.1038/srep14761 (PMC4593005; doi:10.1038/srep14761)
Supplement: Supplementary Information [file srep14761-s1.pdf]

Supporting Information for Determination of the hyperfine magnetic field in magnetic carbon-based materials: DFT calculations and NMR experiments

Jair C. C. Freitas[1], W.L. Scopel[1,2], Wendel S. Paz[1], Leandro V. Bernardes[3], Francisco E. Cunha-Filho[3], Carlos Speglich[3], Fernando M. Araújo-Moreira[3], Damjan Pelc[4], Tonči Cvitanić[4], and Miroslav Požek[4,*]

[1]Department of Physics, Federal University of Espírito Santo (UFES), Av. Fernando Ferrari, 514, 29075-910, Vitória, ES, Brazil.
[2]Department of Exact Sciences, Federal Fluminense University, 27255-250, Volta Redonda, RJ, Brazil.
[3]Department of Physics, Federal University of São Carlos (UFSCar), P.O. Box 676, 13565-905, São Carlos, SP, Brazil
[4]Department of Physics, Faculty of Science, University of Zagreb, Bijenička 32, HR-10000, Zagreb, Croatia
[*]mpozek@phy.hr

Sample preparation

A few grams of both powders, CuO (typically 12 g, from Merck, analytical grade) and synthetic graphite (typically 3 g, from Fluka, with granularity < 0.1 mm) were placed at different alumina crucibles in a sealed atmosphere, inside a tube furnace. The reaction vessel was an alumina tube. The reaction took place at 1200 ^{o}C, during 24 h. After the reaction was finished, the CuO in the first container was partially reduced to Cu(0). The graphite material in the second container (which typically had its volume reduced in about 50 % especially in the side closest to the CuO crucible) exhibited two clearly different regions. The upper layer, which was black and opaque, was formed by powder of ferromagnetic graphite. The material from the lower layer was essentially formed by the pristine graphite that did not take part into the reaction. The method is highly reproducible and yields homogeneous powder samples. The sample selected for the NMR experiments showed a well-defined hysteresis loop in a magnetization versus applied magnetic field measurement conducted at low temperature (1.8 K), with coercive field of ca. 500 Oe, clearly indicating its ferromagnetic character (see Fig. S1).

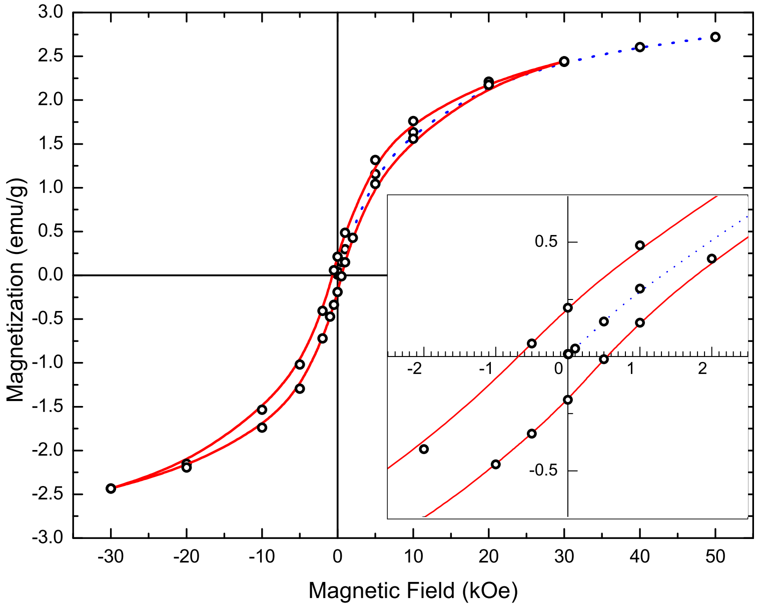

Figure S 1 – Magnetization versus applied magnetic field curve recorded at 1.8 K for the ferromagnetic graphite sample. The inset shows in detail the region close to the origin, from which the coercive field was determined.

Additional NMR data

In order to check the robustness of the sample preparation method, we have measured the zero-field NMR spectrum of a second ferromagnetic graphite sample from another batch. The spectrum is shown in Fig. S2 showing the maximum at nearly the same frequency as for the first sample. This is clear indication of the repeatability of sample preparation, and further evidence of the intrinsic character of the detected zero-field NMR signal.

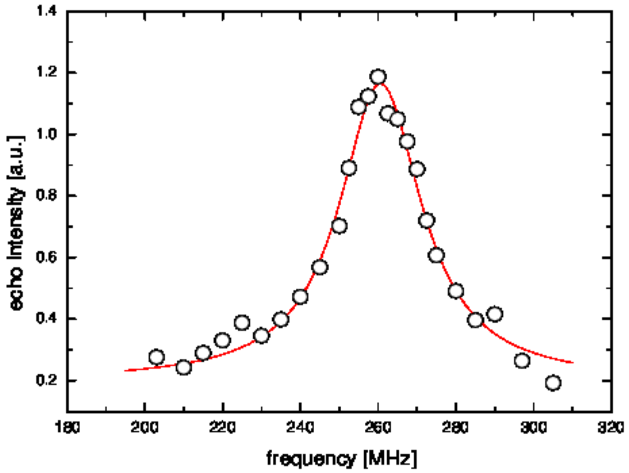

Figure S 2 – Zero-field ^{13}C NMR spectrum recorded at 1.7 K for a second ferromagnetic graphite sample, produced using the same method as the one used for the first sample, but from another batch. This spectrum is to be compared to the one shown in Fig. 3b of the paper.

NMR enhancement

Enhancement of NMR signal intensity in ferromagnets is a well-known consequence of the strong interaction of electronic and nuclear magnetic moments in the material. The nuclei see an effective radio-frequency field amplified by the electronic magnetism. The enhancement effect is significantly larger in domain walls than in the bulk of magnetic domains.[2] Since our experiments are on a randomly oriented powder, to obtain the dependence of NMR spin echo amplitude on the RF field B_1 we need to sum the contributions from domain walls of all orientations with respect to B_1. Also, assumptions have to be made about the dynamics of the wall, i.e. the way it deforms upon the application of B_1. The usual choice is the 'drumhead' model, assuming that the domain walls resemble drumheads pinned at the rim. The echo amplitude in this model[1] is shown to be

$$S \sim \int_0^\infty \int_0^1 \eta(z,x) \ln^2 1/z \sin^3 \gamma\eta(z,x) B_1 \, dz dx \tag{1}$$

with $\eta(z,x) = \eta_0 z/\cosh x$ the distribution of enhancement within the domain wall and at different angles to B_1, η_0 the 'bare' enhancement factor, γ the gyromagnetic ratio, x the dimensionless coordinate along the domain wall profile, and z a derived dimensionless variable. We note that the factor $\sin^3 \gamma\eta(z,x)B_1$ is appropriate for our experimental conditions (two pulses, with the second pulse twice as long as the first), and for other pulse sequences it has a slightly different form. The formula (1) describes the enhancement of NMR signals in iron, nickel, cobalt and many alloys with high accuracy. However, a comparison to the data in ferromagnetic graphite is not entirely satisfactory, especially for high values of B_1 (dotted line in Fig. 3b inset of the main paper – with $\eta_0 = 800$). A different model was thus tried, in the hope that it would better describe the strongly disordered and anisotropic graphite-based material. We assume that there is a strongly preferred direction for domain wall motion, and take the walls to move as a whole (in contrast to the drumhead model, where the rim is pinned). The echo amplitude is then

$$S \sim \int_0^\infty \int_0^\pi \eta(x,\phi) \sin\phi \sin^3 \gamma\eta(x,\phi) B_1 \, d\phi dx \tag{2}$$

with $\eta(z,x) = \eta_0 f(\phi)/\cosh x$, ϕ the angle between the wall and B_1, and $f(\phi)$ a suitable function describing the anisotropy. In our case we employed a Gaussian distribution of the form $f(\phi) = \exp\left(-\sin^2(\phi - \pi/2)/w^2\right)$ with $w \sim 0.1$. The intrinsic

enhancement used in obtaining the solid line in Fig. 3b in the main paper is $\eta_0 = 3000$. The high-B_1 region is better described by the anisotropic model, but the overall agreement with experiment is somewhat worse, and the shape of the curve is still not described well. The domain wall dynamics is thus evidently intricate beyond the simple models employed here; this is perhaps unsurprising, given the microscopic complexity of the material.

References

1. Stearns, M. B. Spin-echo and free-induction-decay measurements in pure Fe and Fe-rich ferromagnetic alloys: domain wall dynamics. *Phys. Rev.* **162**, 496-509 (1967).
2. Turov, E. A. and Petrov, M. P., *Nuclear Magnetic Resonance in Ferro- and Antiferromagnets* (Halsted Press, London, 1972).
